# Effect of External Vibration on PZT Impedance Signature

**DOI:** 10.3390/s8116846

**Published:** 2008-11-01

**Authors:** Yaowen Yang, Aiwei Miao

**Affiliations:** School of Civil and Environmental Engineering, Nanyang Technological University, 50 Nanyang Avenue, Singapore 639798; E-Mail: miao0009@ntu.edu.sg

**Keywords:** PZT, electromechanical impedance, structural health monitoring, vibration

## Abstract

Piezoelectric ceramic Lead Zirconate Titanate (PZT) transducers, working on the principle of electromechanical impedance (EMI), are increasingly applied for structural health monitoring (SHM) in aerospace, civil and mechanical engineering. The PZT transducers are usually surface bonded to or embedded in a structure and subjected to actuation so as to interrogate the structure at the desired frequency range. The interrogation results in the electromechanical admittance (inverse of EMI) signatures which can be used to estimate the structural health or integrity according to the changes of the signatures. In the existing EMI method, the monitored structure is only excited by the PZT transducers for the interrogating of EMI signature, while the vibration of the structure caused by the external excitations other than the PZT actuation is not considered. However, many structures work under vibrations in practice. To monitor such structures, issues related to the effects of vibration on the EMI signature need to be addressed because these effects may lead to misinterpretation of the structural health. This paper develops an EMI model for beam structures, which takes into account the effect of beam vibration caused by the external excitations. An experimental study is carried out to verify the theoretical model. A lab size specimen with different external excitations is tested and the effect of vibration on EMI signature is discussed.

## 1. Introduction

The EMI method has emerged as a widely recognized technique for dynamic identification and health monitoring of structural systems. The electromechanical admittance response of the smart system is derived from the dynamic interaction between the PZT transducer and the host structure. The EMI method has been proven direct and easy to implement. The method is typically applied using an electrical impedance analyzer, which scans a predetermined frequency range band in the order of tens to hundreds of kHz. In doing so, the complex admittance or impedance spectrum may be recorded and meaningful information containing the physical properties of the structure may be extracted. For SHM applications, these spectra can be compared at various times during the service lifespan of the structure, with which any change between the spectra is an indication of the presence of damage or material deterioration.

Essentially the EMI method is founded upon the wealth of research accumulated from the modeling studies on electromechanical interaction between the structure and the PZT transducer, which started about two decades ago. Liang *et al.* [1] first proposed the EMI modeling approach for solving practical problems involving PZT actuator-structure interaction. This one-dimensional model has subsequently been developed and extended to two-dimension by other researchers [2–6]. The method was originally employed for the identification of structural parameters [7] and the design of piezoceramic driven electromechanical systems [8]. Sun *et al.* [9] first applied the EMI concept for the damage detection of a lab size truss structure, followed by Ayres *et al.* [10] who used the method for the detection of loosening prototype truss joints. Since then, the EMI based SHM method has been widely applied to various engineering structures, including aerospace [11, 12], civil [13–21] and mechanical [22, 23] structures. The EMI method has also been applied for vibration control [24].

However, the existing EMI method does not consider the structural vibration caused by the external excitation other than the PZT actuation, which means that the existing EMI method is only applicable to the static structures. Unfortunately, many structures are subjected to vibrations in practice. The vibrations of the structures will influence the results of SHM by causing the changes in PZT signatures. The changes in PZT signatures caused by the external excitation and the structural damage must be differentiated such that the correct information of structural health can be obtained. Therefore, this paper studies the effect of vibration on PZT signature by developing a new EMI model for beam structures with external excitations. Furthermore, experimental tests are carried out to verify the developed model.

## 2. EMI Model

As mentioned previously, the EMI method has been applied for the structures without excitations. However, no further research has been conducted for the EMI modeling of structures with excitations. This part will focus on the theoretical derivations of EMI models with excitations including both extensional and transverse vibrations. Uniform beams with simply supported boundary conditions will be considered.

The equation of motion for the extensional vibration subjected to distributed force of an Euler-Bernoulli thin beam is given as:

(1) $$E_{s}A_{s}u^{\prime \prime }-\rho_{s}A_{s}\ddot{u}=f(x,t)$$

where *E_s_* is the elastic modulus; *A_s_* = *h_s_b_s_* is the cross-sectional area of the beam; *ρ_s_* is the material density of the beam; *u*(*x*,*t*) is the extensional displacement of the beam; *f*(*x*,*t*) is the harmonic distributed force; and *b_s_* and *h_s_* are the width and height of the beam, respectively. Assuming that both of the displacement and distributed force are variable separable:

(2) $$u(x,t)=U(x)e^{i\omega t};f(x,t)=\hat{f}(x)e^{i\omega t};\text{and}\;\hat{f}(x)=\overset{\infty }{\underset{k=1}{\text{∑}}}F_{k}\cos\frac{k\pi }{l_{s}}x$$

where $F_{k}=\frac{2}{l_{s}}\int_{0}^{l_{s}}\hat{f}(x)\cos\frac{k\pi }{l_{s}}\text{xdx}$; *ω* is the frequency of external excitation; and *l_s_* is the length of the beam.

For a simply supported beam whose strains at two ends are zero, the boundary conditions are:

(3) $$u\prime (0,t)=0\;\text{and}\;u\prime (l_{s},t)=0$$

From Eqs.(1) – (3), the extensional displacement for the forced vibration of the simply supported beam can be obtained as:

(4) $$u(x,t)=\overset{\infty }{\underset{k=1}{\text{∑}}}\frac{F_{k}}{\rho_{s}A_{s}[\omega^{2}-{\left(\frac{k\pi }{l_{s}}\right)}^{2}\frac{E_{s}}{\rho_{s}}]}\cos\frac{k\pi }{l_{s}}xe^{i\omega t}$$

The equation of motion for the transverse vibration of the same beam is given as:

(5) $$E_{s}I_{s}w^{\text{iv}}+\rho_{s}A_{s}\ddot{w}=g(x,t)$$

where $I_{\text{s}}=h_{s}^{3}b_{s}/12$ is the moment of inertia of the beam; *w*(*x*,*t*) is the transverse displacement of the beam; the superscript *iv* means the fourth derivative with respect to *x*; and *g*(*x*,*t*) is the distributed force. Assuming that both of the displacement and distributed force are variable separable:

(6) $$w(x,t)=W(x)e^{i\omega t};g(x,t)=\hat{g}(x)e^{i\omega t}\;\text{and}\;\hat{g}(x)=\overset{\infty }{\underset{k=1}{\text{∑}}}G_{k}\sin\frac{k\pi }{l_{s}}x$$

where $G_{k}=\frac{2}{l_{s}}\int_{0}^{l_{s}}\hat{g}(x)\sin\frac{k\pi }{l_{s}}\text{xdx}$.

For a simply supported beam whose displacements and bending moments at two ends are zero, the boundary conditions are:

(7) $$w(0,t)=0;w^{\prime \prime }(0,t)=0;w(l_{s},t)=0;\;\text{and}\;w^{\prime \prime }(l_{s},t)=0$$

From Eqs. (5) – (7), the transverse displacement for the forced vibration of the simply supported beam can be obtained as:

(8) $$w(x,t)=\overset{\infty }{\underset{k=1}{\text{∑}}}\frac{G_{k}}{\rho_{s}A_{s}[{\left(\frac{k\pi }{l_{s}}\right)}^{4}\frac{E_{s}I_{S}}{\rho_{s}A_{s}}-\omega^{2}]}\sin\frac{k\pi }{l_{s}}xe^{i\omega t}$$

Based on small displacement assumption, the following displacement field is defined for the extensional displacement of a generic point on the surface of the beam:

(9) $$u_{s}(x,t)=u(x,t)-\frac{h_{s}}{2}w^{\prime }(x,t)$$

Therefore, the total extensional displacement is:

(10) $$u_{s}(x,t)=\overset{\infty }{\underset{k=1}{\text{∑}}}\frac{F_{k}}{\rho_{s}A_{s}[\omega^{2}-{\left(\frac{k\pi }{l_{s}}\right)}^{2}\frac{E_{s}}{\rho_{s}}]}\cos\frac{k\pi }{l_{s}}xe^{i\omega t}-\frac{kh_{s}\pi }{2l_{s}}\overset{\infty }{\underset{k=1}{\text{∑}}}\frac{G_{k}}{\rho_{s}A_{s}[{\left(\frac{k\pi }{l_{s}}\right)}^{4}\frac{E_{s}l_{s}}{\rho_{s}A_{s}}-\omega^{2}]}\cos\frac{k\pi }{l_{s}}xe^{i\omega t}$$

**Figure 1.**
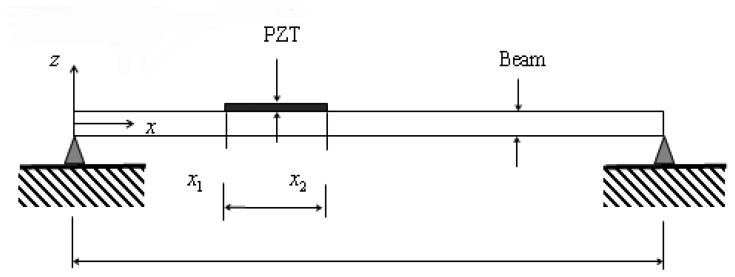
A simply supported beam bonded with a PZT transducer.

Hence, based on response compatibility, the total induced displacement of the PZT transducer is equal to the displacement difference between two discrete points on the surface of the beam, coincident to the ends of the transducer:

(11) $$u_{\text{PZT}}=u_{s}(x,t){|}_{x_{2}}-u_{s}(x,t){|}_{x_{1}}$$

where *x*_1_ and *x*_2_ are the coordinates of the two ends of the PZT transducers, as shown in Figure 1.

Consequently, the total displacement of the PZT transducer is:

(12) $$u_{\text{PZT}}\left(x,t\right)=\sum_{\text{k}=1}^{\infty }\left\{\frac{F_{k}}{\rho_{s}A_{s}\left[\omega^{2}-{\left(\frac{k\pi }{l_{s}}\right)}^{2}\frac{E_{s}}{\rho_{s}}\right]}-\frac{kh_{s}\pi }{2l_{s}}\frac{G_{k}}{\rho_{s}A_{s}\left[{\left(\frac{k\pi }{l_{s}}\right)}^{4}\frac{E_{s}I_{s}}{\rho_{s}A_{s}}-\omega^{2}\right]}\right\}\left[\cos\frac{k\pi }{l_{s}}x_{2}-\cos\frac{k\pi }{l_{s}}x_{1}\right]e^{i\omega t}$$

The following is the displacement of PZT vibration subjected to an electric potential:

(13) $$u_{\text{pzt}}\left(x,t\right)\left[\sum_{n=1}^{\infty }\frac{F_{n}\left(\phi_{n}\left(x_{2}\right)-\phi_{n}\left(x_{1}\right)\right)}{\rho_{s}A_{s}\left(\Phi_{n}^{2}-{\omega_{\text{PZT}}}^{2}\right)}+\frac{h_{s}}{2}\sum_{m=1}^{\infty }\frac{F_{m}\left(\varphi_{m}^{'}\left(x_{1}\right)-\varphi_{m}^{'}\left(x_{2}\right)\right)}{\rho_{s}A_{s}\left(\Omega_{m}^{2}-{\omega_{\text{PZT}}}^{2}\right)}\right]e^{i\omega_{{\text{PZT}}^{t}}}$$

where *ω_PZT_* is the frequency of PZT actuation; $\phi_{n}\left(x\right)=\cos\left(\frac{n\pi x}{l_{s}}\right)$; $\Phi_{n}=\frac{n\pi }{l_{s}}\sqrt{\frac{E_{s}}{\rho_{s}}}$; $F_{n}=Q_{u}^{-1}{\hat{F}}_{\text{PZT}}\left[\phi_{n}\left(x_{2}\right)-\phi_{n}\left(x_{1}\right)\right]$; $\varphi_{m}\left(x\right)=\sin\left(\frac{m\pi x}{l_{s}}\right)$; $\Omega_{m}={\left(\frac{m\pi }{l_{s}}\right)}^{2}\sqrt{\frac{E_{s}I_{s}}{\rho_{s}A_{s}}}$; $F_{m}=Q_{w}^{-1}\frac{h_{s}}{2}{\hat{F}}_{\text{PZT}}\left[\varphi_{m}^{'}\left(x\right){|}_{x=x_{1}}-\varphi_{m}^{'}\left(x\right){|}_{x=x_{2}}\right]$; *Q_u_* = *Q_w_* = 0.5*l_s_*; and *F̂_PZT_* is the magnitude of PZT actuation force. Detailed derivations of Eq.(13) can be found in [23].

Finally, we can obtain the electromechanical admittance of the PZT transducer:

(14) $$Y=\frac{I}{V}=\frac{{\tilde{Y}}_{11}^{E}\text{db}}{h}\left\{\frac{\frac{d}{\psi }\sin\left(\gamma l\right)i\omega_{\text{PZT}}+\frac{d}{K_{\text{PZT}}l}\sin^{2}\left(\gamma l\right)\sum_{k=1}^{\infty }\vartheta \left(k\right)\chi \left(k\right)e^{i\left(\omega -\omega_{\text{PZT}}\right)t}i\left(\omega -\omega_{\text{PZT}}\right)}{\psi^{2}{\left\{\sum_{n=1}^{\infty }\delta \left(n\right)+\frac{h_{s}}{2}\sum_{m=1}^{\infty }\zeta \left(m\right)+\sum_{k=1}^{\infty }\left\{\vartheta \left(k\right)\right\}\chi \left(k\right)e^{i\left(\omega -\omega_{\text{PZT}}\right)t}\right\}}^{2}}\right\}+\frac{\tilde{\varepsilon }i\omega_{\text{PZT}}\text{lb}}{h}$$

where:

$$\begin{array}{l} \psi =\gamma \cos\left(\gamma l\right)+\frac{R}{l}\sin\left(\gamma l\right);\\ \varsigma =\rho_{s}A_{s}{\hat{F}}_{\text{PZT}};\\ \vartheta \left(k\right)=\frac{F_{k}}{\varsigma \left[\omega^{2}-\Phi_{k}^{2}\right]}-\frac{kh_{s}\pi }{2l_{s}}\frac{G_{k}}{\varsigma \left[\Omega_{k}^{2}-\omega^{2}\right]};\\ \chi \left(k\right)=\cos\frac{k\pi }{l_{s}}x_{2}-\cos\frac{k\pi }{l_{s}}x_{1};\\ \delta \left(n\right)=\frac{F_{n}\left(\phi_{n}\left(x_{2}\right)-\phi_{n}\left(x_{1}\right)\right)}{\varsigma \left(\Phi_{n}^{2}-{\omega_{\text{PZT}}}^{2}\right)};\\ \zeta \left(m\right)=\frac{F_{m}\left(\varphi_{m}^{\prime }\left(x_{1}\right)-\varphi_{m}^{\prime }\left(x_{2}\right)\right)}{\varsigma \left(\Omega_{k}^{2}-{\omega_{\text{PZT}}}^{2}\right)}; \end{array}$$

${\tilde{Y}}_{11}^{E}$ is the complex Young's modulus; *d* is the piezoelectric strain coefficient; *γ* is the wave number; *R* = *K_str_K_PZT_*^−1^ is the stiffness ratio; *K_str_* is the dynamic stiffness of the beam structure; $K_{\text{PZT}}={\tilde{Y}}_{11}^{E}\text{bh}l^{-1}$ is the quasi-static stiffness of the PZT transducer; *ε̃* is the complex permittivity of PZT; and *l*, *b* and *h* are the length, width and height of the PZT transducer, respectively.

## 3. Experimental Setup

**Figure 2.**
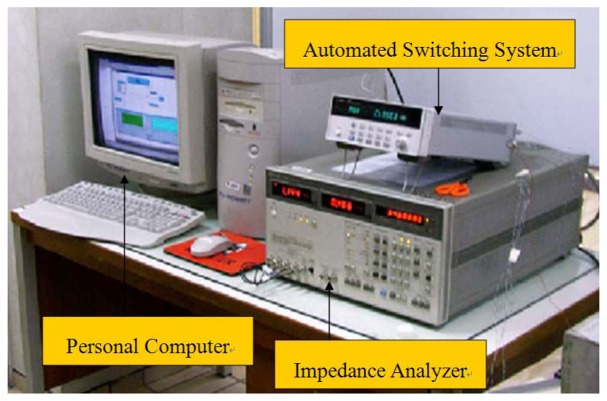
EMI measurement system.

**Figure 3.**
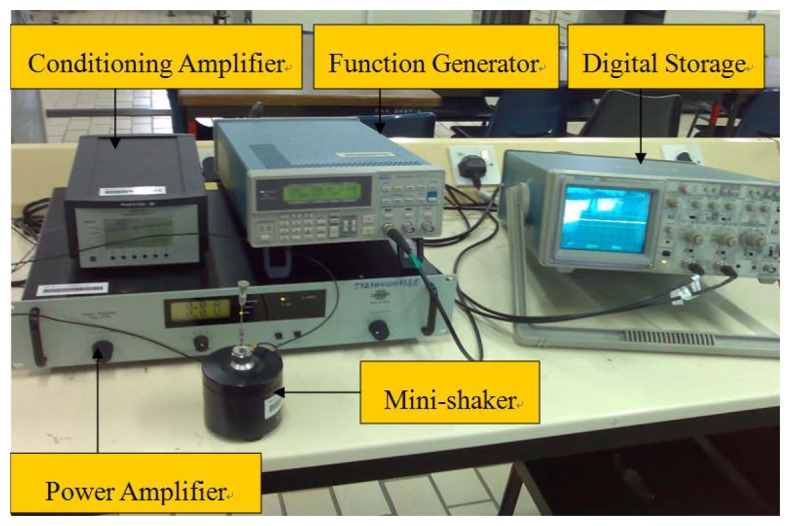
External excitation system.

In order to verify the developed theoretical model, an experimental test is carried out on an aluminum beam bonded with one PZT transducer. The instruments and equipments used in this experiment are shown in Figures 2 and 3.

The HP4192A Analyzer [25] is used to actuate the PZT transducer and simultaneously record its admittance signature. If multiple PZT sensors are used, a switch box is needed to make a multiple connections between the Analyzer and the PZT sensor. For this study, only one channel of the switch box is used since one PZT sensor is used in the experiment. In order to facilitate the automation of the testing, the program VEE Pro v.6.01 is used to control the Analyzer via a GPIB interface card installed in the PC. The scanning frequency range and resolution will be set with the Analyzer to record the PZT admittance signature.

**Figure 4.**
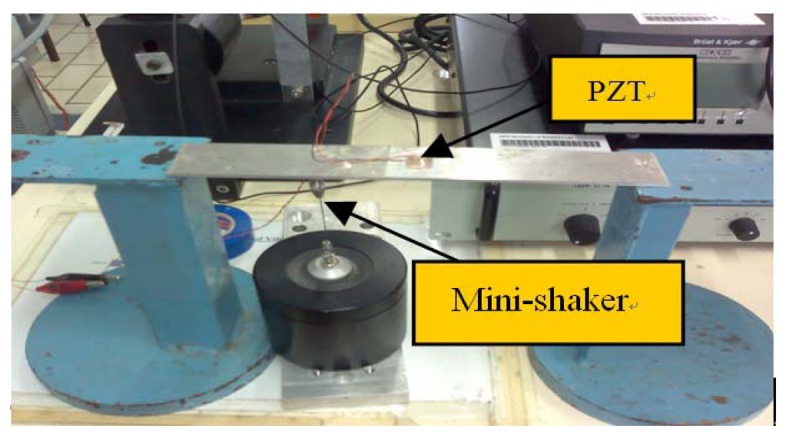
Beam specimen with PZT transducer and mini-shaker

The simply supported beam specimen is shown in Figure 4. The beam is stuck on the platforms by the blue tack. The mini-shaker generates external excitation to the beam at the location of 6cm from the left end of the beam. The PZT transducer is bonded to the beam at the central location. The properties of the PZT transducer and the beam specimen are listed in Tables 1 and 2, respectively.

## 4. Results and Discussion

As presented in the previous part, the theoretical derivation has been conducted to obtain the final result of the electromechanical admittance for a simply supported beam. And for practical cases, the transverse vibration of the beam is much more critical compared with the extensional vibration. Therefore, only the transverse vibration is considered in the experimental test. Then the electromechanical admittance of PZT can be simplified from Eq. (14) as:

(15) $$Y=\frac{I}{V}=\frac{{\hat{Y}}_{11}^{E}\text{db}}{h}\left\{\frac{d}{\psi }\sin\left(\gamma l\right)i\omega_{\text{PZT}}-\frac{\frac{d}{K_{\text{PZT}}}\sin^{2}\left(\gamma l\right){K_{\text{str}}}^{2}}{\psi^{2}}\left\{\frac{h_{s}\pi }{2l_{s}l\varsigma }\sum_{k=1}^{\infty }k\chi \left(k\right)\left[\frac{G_{k}}{\Omega_{k}^{2}-\omega^{2}}e^{i\left(\omega -\omega_{\text{PZT}}\right)t}i\left(\omega -\omega_{\text{PZT}}\right)\right]\right\}-\text{ldi}\omega_{\text{PZT}}\right\}+\frac{\tilde{\varepsilon }i\omega_{\text{PZT}}lb}{h}$$

The above equation is applied to obtain the electromechanical admittance over the range of frequency bands. After substituting the values of various parameters into the theoretical formulation and a series of calculations by means of Matlab program, the theoretical results can be obtained. With the change in frequency and magnitude of the excitation force, the electromechanical admittance will change accordingly. Therefore, we will discuss the influence of these two factors.

**Figure 5.**
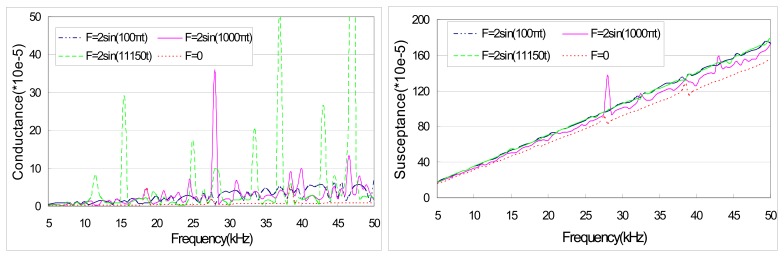
Theoretical prediction of PZT admittance for different excitation frequencies.

Figure 5 shows the theoretical prediction of PZT admittance for external excitations with the same magnitude but different frequencies, which are indicated at the bottom of the diagram. It is apparent that the conductance and susceptance of the PZT sensor are significantly affected by the excitation frequency. With the variations in frequency, both the peak location and the peak magnitude of the PZT admittance signature change. This result indicates that the frequency of external excitation is a critical parameter that influences the PZT signature.

**Figure 6.**
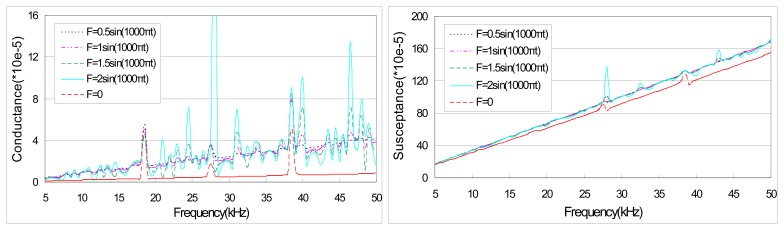
Theoretical prediction of PZT admittance for different excitation magnitudes.

Figure 6 illustrates the theoretical prediction of PZT admittance for external excitations with the same frequency but different magnitudes. It can be observed that both the conductance and susceptance of the PZT sensor do not change drastically for different magnitudes of excitation force. Especially, close inspection shows that the peak locations of PZT admittance are almost the same for different magnitudes of excitation. Moreover, with the increase of magnitude in excitation, the peak magnitude also increases. This result indicates that the magnitude of excitation force has significant impact on the peak magnitude but its effect on the peak location is negligible.

**Figure 7.**
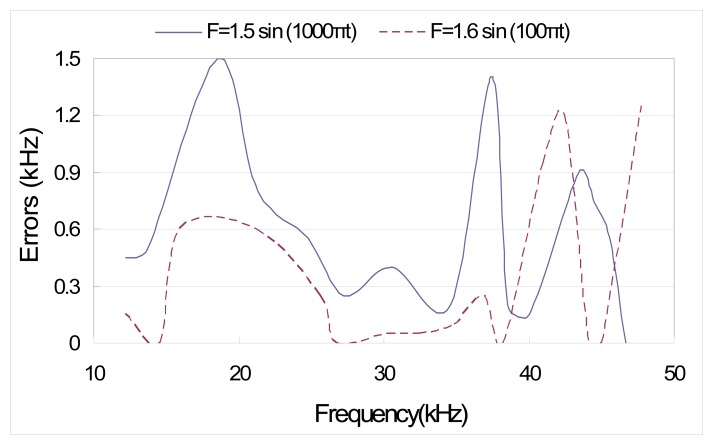
Errors between theoretical and experimental results.

To verify the developed EMI model, we first compared the predicted peak locations with the experimental ones. The peak locations for two different excitations are listed in Table 3. The differences between theoretical predictions and experimental results can be ascribed to several model assumptions as well as experimental errors. One of the major reasons for discrepancies could be that the effect of bonding layer (epoxy) between PZT and structure is not considered in the theoretical modeling. The errors between theoretical predictions and experimental results are also shown in Figure 7. Overall, the accuracy of the theoretical model is acceptable.

**Figure 8.**
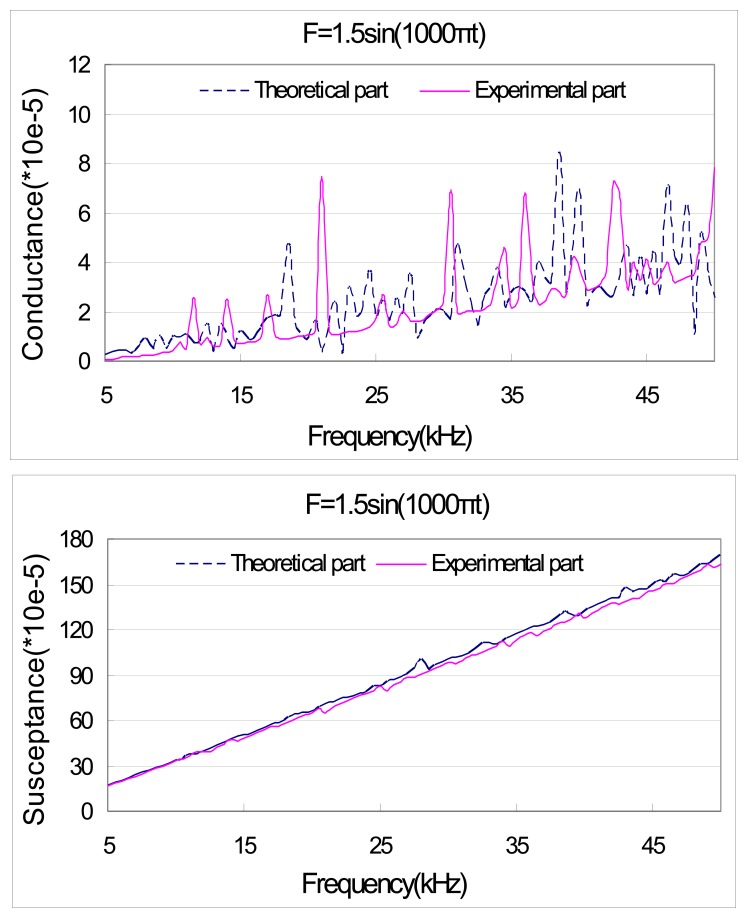
Theoretical and experimental result of PZT admittance with external force F=1.5sin (1000πt).

**Figure 9.**
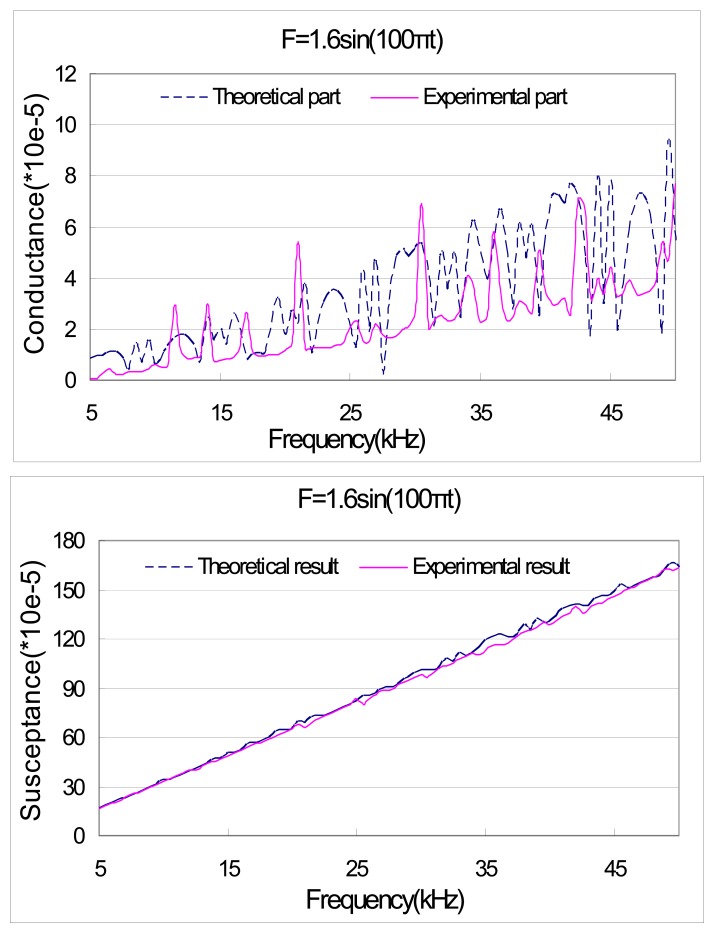
Theoretical and experimental result of PZT admittance with external force F=1.6sin (100πt).

Figures 8 and 9 illustrate the theoretical and experimental admittance signatures. The comparisons show that the theoretical model is capable of predicting fairly accurately some of the electromechanical resonant peaks that can be found in the measured response. Nonetheless, discrepancies exist between the predicted response and the measured response. The most obvious discrepancy is found in the magnitude of the peaks. This discrepancy may be attributable to the simplified one-dimensional nature of the PZT transducer, as well as the assumptions made in the derivation of a closed-form solution for the dynamics of the structural substrate. Again, the accuracy of theoretical model is acceptable.

**Figure 10.**
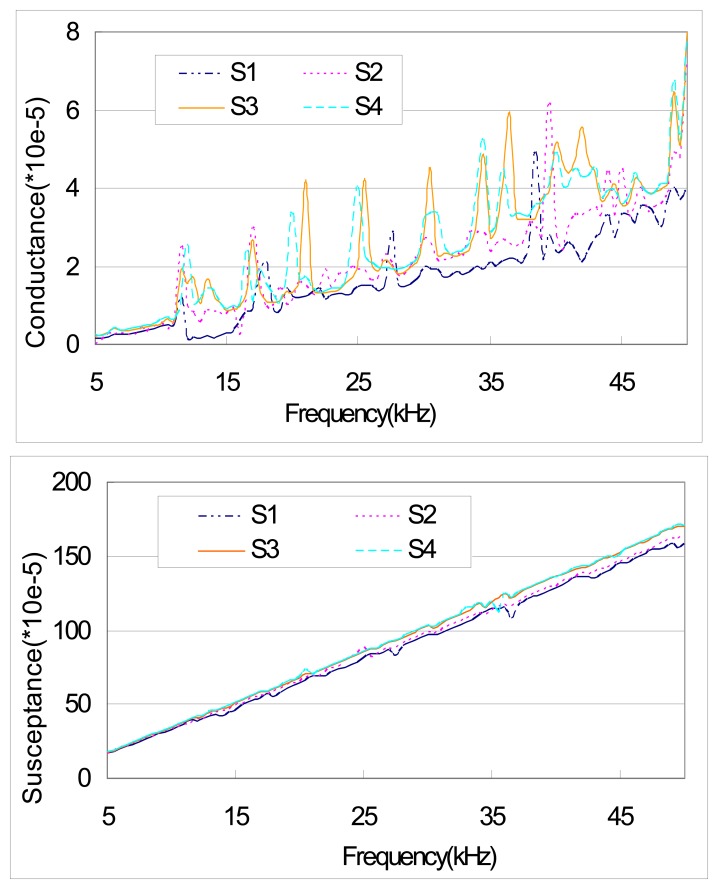
Experimental result of PZT admittance for four cases (S1–S4).

In addition, we introduce damage in the specimen in the experimental test. This attempt is made to compare the peak shifts caused by damage and those caused by external excitation. The conductances for four cases (S1, S2, S3 and S4) are shown in Figure 10. S1 and S2 represent the signatures of the specimen without damage while S3 and S4 represent those with damage. The damage in the specimen is induced by drilling a hole of diameter 5mm with distance of 4cm from the right end of the beam. There is no external excitation applied to the specimen for S1 and S3 while the external excitation (F=sin (1000t)) is applied for S2 and S4. As expected, the shifts of the peaks are obvious (last 3 rows of Table 4). Comparison between S1 and S2 shows the effect of external excitation. From Figure 10 and Table 4, this effect is apparent and there is no consistent shifting trend in peaks. The peak shifts between S1 and S3, and S2 and S4 represent the effect of damage on the signature. From Figure 10 and Table 4 (the last two rows), we can observe that the peak shifts due to damage are comparable for the specimen with or without external excitation. We can also see that the peak shifts due to damage and those due to external excitation are in the same order, implying that the effect of external excitation (vibration) on PZT admittance signature could not be neglected. For damage assessment, this effect should be eliminated; otherwise, it may lead to misinterpretation of the structural damage. Further study on how to eliminate/minimize the effect of vibration is definitely needed, which will be our future work.

**Figure 11.**
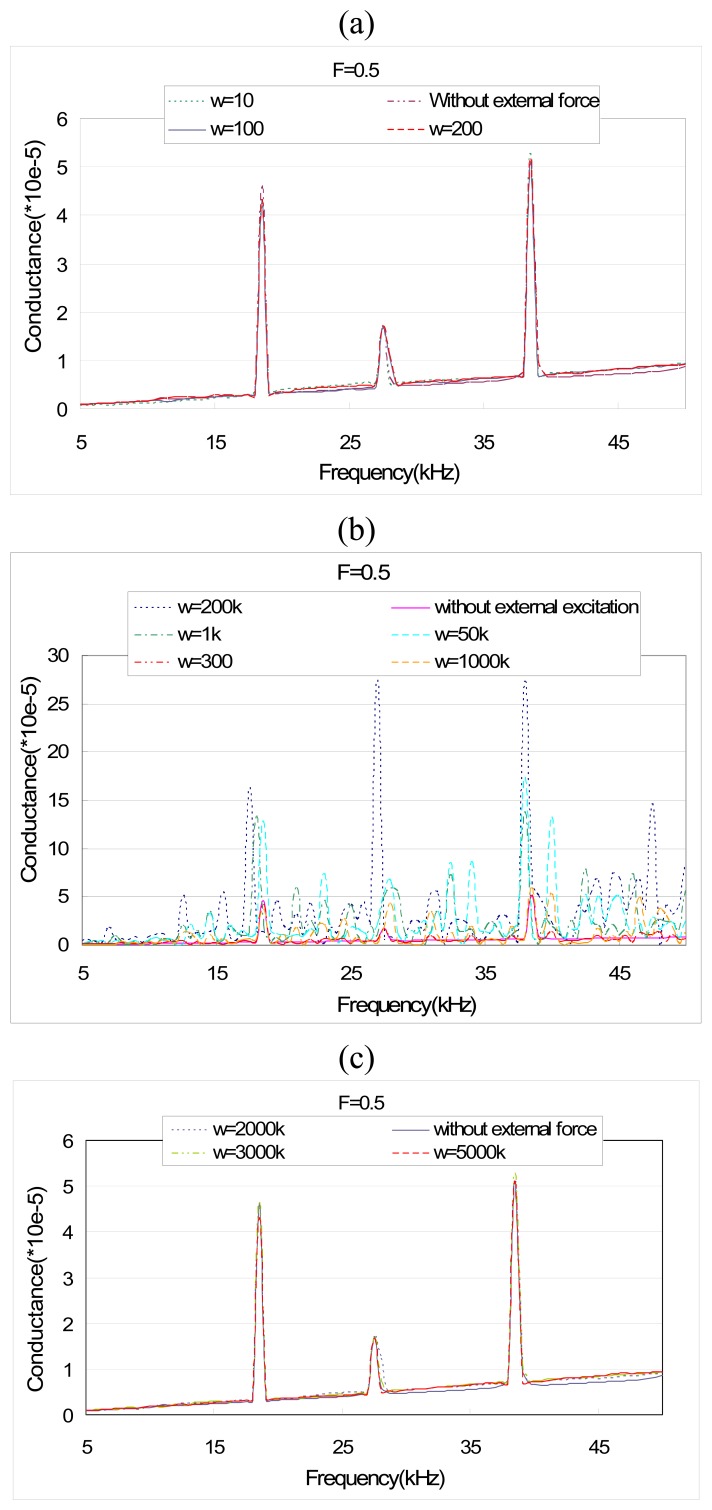
Theoretical prediction of PZT admittance for different excitation frequencies (F=0.5sin*w*t).

Figure 11 illustrates the effect of a broad band of frequency of external vibration on the conductance signature. The results are obtained using the EMI model developed in this paper. From Figure 11(a) where the external frequency *ω* is much smaller than the PZT scanning frequency *ω_PZT_* (5–50 kHz), *ω*/*ω_PZT_* ≤ 1/25, it can be observed that there is almost no peak shift in conductance signature. Similarly, if *ω* is much larger than *ω_PZT_*, e.g., *ω*/*ω_PZT_* ≥ 40, the peak shift in conductance is also negligible, as shown in Figure 11(c). However, for 1/17 ≤ *ω*/*ω_PZT_* ≤ 20 shown in Figure 11(b), obvious peak shifts can be observed. Together with other tries using the theoretical model, we conclude that if the external frequency is much smaller (e.g., *ω*/*ω_PZT_* ≤ 1/25 ) or larger than (e.g., *ω*/*ω_PZT_* ≥ 30 ) the PZT frequency, the influence of external vibration on the EMI signature can be ignored. Otherwise, the effect of external vibration has to be considered in the EMI modeling.

It is worth mentioning that we have focused on the peak location and peak shift because in the EMI technique, peak locations represent the natural frequencies of the host structure. Therefore peak shifts in admittance signature indicate the change of structural properties, which may be caused by damage or vibration. Other statistical method such as the root mean square deviation (RMSD) can also be used to analyze the signature [18].

## 4. Conclusions

Although piezoelectric material has been successfully used for structural damage detection in many areas, the influence of the external excitations on the signature of PZT needs further study. This paper aims at ascertaining the effect of external excitation (vibration) on the PZT signature. The fundamental formulation of EMI model is first derived for a simply supported beam with external excitations. In the experiments, an aluminum beam specimen bonded with a PZT sensor is tested. The admittance signatures of the PZT sensor are measured for various excitations. The experimental results are compared with the theoretical predictions to investigate the reliability of the model. Overall, the theoretical model is capable of predicting the resonant peaks in the admittance signature. Further experimental tests are carried out to compare the effects of excitation and damage on the admittance signature. And the effective frequency range of external excitation is studied by the developed model. It is concluded that the effect of external excitation (vibration) on PZT admittance signature could not be neglected if the external excitation frequency and PZT's actuation frequency are comparable. For structural damage assessment, further study on how to eliminate this effect is needed.

## Figures and Tables

**Table 1.** Properties of PZT transducer.

**Young's Modulus*Y*(*GPa*)**	**Loss Factor**	**Mass Density** *ρ*(*kg*/*m*^3^)	**Strain Constant d** *d*_31_**(*m*/*volt*)**	**Permittivity $\varepsilon_{33}^{T}$ (*farad*/*m*)**	**Dielectric Loss Factor***δ*	**Dimension (*mm*)**	**Location (*mm*)**
68.9	0.001	7800	-2.10E-10	2.36E-08	1.47E-02	*l*=*b*=10 *h*=0.2	100 *mm* from both ends

**Table 2.** Properties of beam specimen.

**Length (*mm*)**	**width (*mm*)**	**Thickness (*mm*)**	**Thickness Young's Modulus (*GPa*)**	**Poisson's Ratio**	**Mass Density** (*kg* / *m*^3^ )	**Damping Ratio**
200	30	2	68.9	0.3	2700	0.005

**Table 3.** Theoretical and experimental peak locations for F=1.5sin (1000πt) and F=1.6sin (100πt).

**F=1.5 sin (1000πt)**			**F=1.6 sin (100πt)**		

**Peak locations (kHz)**			**Peak locations (kHz)**		

Theoretical predictions	Experimental results	Errors	Theoretical predictions	Experimental results	Errors

12.25	11.80	-0.45	12.10	11.95	-0.15
13.75	14.25	+0.50	14.60	14.60	0
18.50	17.00	-1.50	15.90	16.50	+0.60
21.30	20.50	-0.80	21.15	20.55	-0.60
24.75	25.20	+0.55	25.60	25.35	-0.25
27.25	27.00	-0.25	26.70	26.70	0
30.55	30.15	-0.40	30.25	30.30	+0.05
34.55	34.75	+0.20	32.40	32.35	-0.05
37.40	36.00	-1.40	34.85	34.75	-0.10
38.50	38.30	-0.20	36.90	36.65	-0.25
40.00	39.85	-0.15	38.25	38.25	0
43.40	42.50	-0.90	40.95	40.00	-0.95
44.50	43.75	-0.75	42.45	43.65	+1.20
45.55	45.00	-0.55	44.10	44.10	0
46.70	46.70	0	45.00	45.00	0
			47.75	46.50	-1.25

**Table 4.** Peak locations and shifts [unit: kHz].

**Cases**	**Peak locations and peak shifts**								

S1	11.5	18	22	27.5	30	34	35	38.5	41
S2	11.5	17	21.5	27	30	33.5	35.5	39.5	42.5
S3	12.5	17	21	25.5	30.5	34.5	36.3	39.5	42
S4	12.3	16.2	20.2	25	30.5	34.2	36.3	40.2	43.5
S2–S1	0	-1	-0.5	-0.5	0	-0.5	+0.5	+1	+1.5
S3–S1	+1	-1	-1	-2	+0.5	+0.5	+1.3	+1	+1
S4–S2	+0.8	-0.8	-1.3	-2	+0.5	+0.7	+0.8	+0.7	+1
